# Molecular and bacteriological investigations for the co-existence CRISPR/Cas system and β-lactamases of types extended-spectrum and carbapenemases in Multidrug, extensive drug and Pandrug-Resistant *Klebsiella pneumoniae*

**DOI:** 10.1016/j.sjbs.2024.104022

**Published:** 2024-05-19

**Authors:** Hekmat A. Owaid, Mushtak T.S. Al-Ouqaili

**Affiliations:** a Department of Biology, College of Science, University of Anbar, Ramadi, Iraq; b Department of Microbiology, College of Medicine, University of Anbar, Al-Anbar Governorate, Ramadi, Iraq

**Keywords:** ESBLs, Carbapenemases, CRISPR/Cas, *K. pneumoniae*, Antibiotic resistance

## Abstract

The recent approach towards combating the antimicrobial resistance has led to the use of Clustered Regularly Interspaced Short Palindromic Repeats (CRISPR) and associated sequence to overcome the challenges of antimicrobial resistance. Thus, this study aimed to detect the underlying resistance mechanisms such as ESBLs and carbapenemases and whether there is a correlation between multidrug, extensive drug and pan drug resistance and the occurrence of CRISPR loci. A total of one hundred study isolates were subjected to antimicrobial susceptibility testing using the AST card of the Vitek technique to detect resistance patterns involving ESBLs and carbapenemase (CRE). An investigation of the genes encoding CRISPR/Cas systems using PCR was achieved. Out of 81 (81.0%) resistant *Klebsiella pneumoniae isolates*, 71 (71%) and 21 (21.0%) produced ESBLs and carbapenemases, respectively. Also, 53 (53.0%), 19 (19.0%) and 9 (9.0%) were MDR, XDR, and PDR respectively. It was noted that Cas1, Cas3, CRISPR1, CRISPR2 and CRISPR3 were positive in 38 (38.0%) of the isolates, while CRISPR1 for incomplete CRISPR1-Cas systems alone was detected in 78 (78.0%). Further, the number of intact CRISPR1, intact CRISPR2 and intact CRISPR3 types were 7 (27.0%), 34 (34%) and 18 (18.0%) respectively. It is concluded that antibiotic resistance levels were inversely correlated with the existence of CRISPR/Cas systems. The absence of the CRISPR/Cas system increases the prevalence of MDR, XDR and PDR in ESBL and carbapenem-producing *Klebsiella pneumoniae*. With the increase in the degree of antibiotic resistance (MDR, XDR to PDR), the occurrence ratio of the (CRISPR)/CRISPR-associated sequence decreased.

## 1 Introduction

The rapid emergence of superbugs (antimicrobial resistant bacteria) (Aslam et al., 2021) has become the most pressing global public health concern in recent years. One of these superbugs is the gram-negative bacillary-shaped Enterobacterales-order bacterium *Klebsiella pneumoniae*, which includes both XDR and PDR (Sleiman et al., 2021).

Numerous antibiotic resistance genes from *Klebsiella pneumoniae* are present in other Enterobacteriaceae species, mostly through self-transferrable plasmids, which are well recognized to be the main causes of antibiotic resistance in several bacteria (Chinemerem Nwobodo et al., 2022).

The production of carbapenemase enzymes and extended spectrum-lactamase (ESBL) are the major resistance mechanisms which *K. pneumoniae* has developed (Oliveira et al., 2022a). The activity of numerous Beta-lactam antibiotics, such as cephalosporins, penicillins, monobactams, and carbapenems, that are widely prescribed on a global scale, can be inhibited by distinct variants of these enzymes (De Angelis et al., 2020). Infections induced by ESBLs producer isolates of *K. pneumoniae* are conventionally managed with the administration of carbapenem antibiotics. Nevertheless, the occurrence of carbapenemase producer isolates of *K. pneumoniae* has increased in recent period, necessitating the substitution of carbapenems with alternative medications such as tigecycline and colistin. These treatments have become the sole viable therapeutic choice for managing infections caused by XDR bacteria (Al-Ouqaili et al., 2020).

In numerous bacterial species, the presence of CRISPR-Cas serves as a mechanism of defence against foreign genomes, including resistance genes and plasmids (Wu et al., 2021). The CRISPR/Cas system consists of Cas genes located upstream, a collection of foreign DNA sequences referred to as spacers, and direct and inverted repeats of equal size and identity, commonly known as palindromic repeats (Wang et al., 2020). The spacers in question originate from bacterial cell invasion by foreign plasmids and phages; inserting these spacers into the CRISPR array in a series indicates that the bacterial cell has acquired a “memory fragment” of these invaders. By acting as a sequence-specific bacterial defense mechanism, bacteria cleave the DNA sequences corresponding to their spacers by utilizing the nuclease action of Cas proteins (Gholizadeh et al., 2020).

Antibiotic resistance is inversely related to the existence of the CRISPR and associated sequences in the DNA of certain prokaryotes including bacteria, specifically *K. pneumoniae*, according to an enormous amount of research published in the last decade. (Wang et al., 2020). Nevertheless, the findings in this particular domain have occasionally shown conflicting outcomes, hence highlighting the evident necessity for further investigation in this realm (Tao et al., 2022).

Understanding the molecular events in the mechanisms of antimicrobial resistance is a corner stone for overcoming this problem at the core level. Thus, this article aimed to determine the mechanisms of resistance of *K. pneumoniae* to beta-lactams such as ESBLs and carbapenemases and to determine whether there is a correlation between such mechanisms in MDR, XDR and PDR and the occurrence CRISPR-Cas system loci. Gaining a comprehensive understanding of this correlation can yield novel perspectives on potential pharmacological targets aimed at combating resistant infections caused by this bacterium.

## 2 Patients and methods

### 2.1 Ethics statement

The Committee of Medical Ethics of University of Anbar has approved this research with the approval number 14, in March 15, 2022) according to the Declaration of Helsinki. All of the study participants who were patients or their parents provided written informed consent.

### 2.2 The patients and design of study

The cross-sectional survey in this study was achieved in the period from February to October 2022. The patients were admitted to Ramadi Teaching Hospitals, provided a variety of clinical specimens. A total of one hundred distinct isolates of *Klebsiella pneumoniae* were bacteriologically diagnosed in study samples. Six percent of the ear swabs were from patients with otitis media, 17 % were from patients with bronchial infection, 50 % were from catheterized patients, and 27 % were from patients with burns, osteomyelitis, and diabetic foot infections.

### 2.3 Bacteriological identification

The laboratories of microbiology at the same hospitals handled the processing and culture of the clinical specimens and carried out all the bacteriological analyses and confirmatory biochemical testing. The selective culture media which include Blood agar, MacConkey agar, and Eosin Methylene blue agar from Merck Co., Germany were used to cultivate all specimens, and they were incubated for 24 to 48 h at 37 °C. In addition to lysine iron agar, triple sugar iron agar, urea/citrate utilization, ornithine decarboxylase, and Gram stain, the study isolates of *K. pneumoniae* were detected based on cultural and morphological characteristics (Saki et al., 2022)]. The bacterium was identified definitively and confirmatively using VITEK-2 GN ID cards and the VITEK®2 Compact B System which is originated from (BioMérieux, France) (Oliveira et al., 2022).

### 2.4 Antimicrobial susceptibility test

Antimicrobial susceptibility testing of the isolates was conducted using the AST-GN cards of the VITEK®2 Compact B System according to the instructions provided by the manufacturer. The results of antimicrobial susceptibility testing (AST) were obtained for amikacin, ciprofloxacin, tigecycline, nitrofurantoin, cefoxitin, cefazolin, ceftriaxone, ceftazidime, cefepime, imipenem, ertapenem, gentamicin, amikacin, levofloxacin, ciprofloxacin, nitrofurantoin, in addition to tigecycline.

The results are presented using MIC values at which the guidelines of the CLSI (Clinical and Laboratory Standard Institute) were adopted. The samples were classified into susceptible, intermediate, or resistant (Kurumisawa et al., 2021). The (EUCAST), European Committee on Antimicrobial Susceptibility Testing provided the parameters for interpreting tigecycline AST results in 2021 (Gaur et al., 2023). To ensure the quality of the antimicrobial susceptibility test. E. coli ATCC 25922 was used as an internal quality control.

The bacteria were categorized as MDR, which is defined as being resistant to three or more different group of Antimicrobial agents, based on their profiles of antibiotic susceptibility. When isolates were found to be resistant to at least one antibiotic across six different classes, we referred to them as XDR. Finally, if an isolate could withstand every antibiotic that was tested, it was considered to be PDR (Halah and Al-Hasani, 2023).

## 3 Molecular assay

### 3.1 DNA extraction

Following the manufacturer's instructions, The extract ion of the genomic DNA has been done using SaMag-12TM automated DNA extraction system and the SaMag automated bacterial DNA extraction kit (SaMag, Cepheid, Italy). Quantity of the study DNA was detected using a QuantusTM fluorometer (Promega, USA) to evaluate the quality of the sample for potential use in the future. (Hussein et al., 2021).

### 3.2 CRISPR/Cas genes detection

The CRISPR/Cas genes were investigated using specific primers and amplifying the encoding genes using conventional polymerase chain reaction (PCR) as represented in Table 1 (Wang et al., 2020). The initial denaturation of Cas1 and Cas3 was carried out at 95 °C for 5 min, followed by denaturation at 94 °C for 1 min, annealing at 60 °C for 30 s, and extension at 72 °C for 1 min in the PCR cycle. A total of 35 cycles consisting of denaturation, annealing, and extension were conducted. The final extension phase lasted 10 min at 72 °C. For Cas9 and Cse1, gradient PCR was used to vary the annealing temperature within the 54–66 °C range. The PCR cycle for all types of CRISPR followed the same steps, starting with five minutes at 95 °C to denature the DNA. Following denaturation for one minute at 94 °C, the samples were annealed for one minute at 63 °C and extended for one minute at 72 °C. After 35 cycles of denaturation, annealing, and extension, a final extension phase was conducted, which lasted for 10 min at 72 °C. After that, gel electrophoresis with a 1.5 % agarose gel containing 10 % ethidium bromide was used to separate the PCR products. Electrophoresis was performed in 1X Tris/Borate/EDTA (TBE) buffer at 50 V for 5 min and 100 V for 1 h. To ensure that the PCR product band was of the correct size, we compared it to a DNA ladder with 100-base-pair (Fermentas, Maryland, USA). The UV transilluminator with the manufacturing company of Vilber lourmat, Marne-la-Vallée, France was used to detect the fluorescent band after completing agarose gel electrophoresis.

**Table 1:** The gene primers details (names, sequences, purpose, size, guanine: Cytosine % and melting temperature).

**NO.**	**Gene Primers**	**DNA sequence** **(5ʹ→3ʹ)**	**Purpose**	**The size of the Product (bp)**	**GC** **(%)**	**Tm (ͦC)**
**1-**	**Cas 1**	**Forward-**GCTGTTTGTCAAAGTTACCCGCGAACTC	For Cas1 gene	208	50.0	66.7
		**Reverse-**GTTTTGATCGCCTCATGAGTCACAGTTG			48.28	66.2
**2-**	**Cas 3**	**Forward-**GGGTTTCGCTACAAAATCAACATGCCATCG	For Cas3 gene	506	46.6	67.1
		**Reverse-**CACGAGTTTTTTACGCTCATCAAACCAGAGCG			46.8	68.2
**3-**	**Cas 9**	**Forward**- acgccaattggttgaaactc		225	45.0	60.0
		**Reverse**- acgacggcattaagatacgc			50.0	60.0
**4-**	**Cse1**	**Forward**- CAGTTTAACCGATATTTTCAGCCAGCCGG	For Cse1 gene	347	48.2	66.36
		**Reverse**- CATCAGTTAATTGCTGCTGTTGCTGACTTTCG			43.7	67.02
**5-**	**I-E-CR 1**	**Forward**- CTGGCATAACGCCACCGG	For incomplete CRISPR1-Cas systems	898–3700	66.6	61.2
		**Reverse**- GAGACCCGGTTCTTCGGGC			68.4	62.6
**6-**	**I-E-CR1**	**Forward-** CAGTTCCTGCAACCTGGCCT	For intact CRISPR1-Cas systems	Variable	60.0	62.7
		**Reverse-** CTGGCAGCAGGTGATACAGC			60.0	61.09
**7-**	**I-E-CR2**	**Forward**- GCGCTACGTTCTGGGGATG	For incomplete CRISPR2-Cas systems	522–2850	63.16	60.8
		**Reverse**- CGTCGCAAAACTCGACCAGA			55.0	60.9
**8-**	**I-E-CR2**	**Forward-** GTAGCGAAACCCTGATCAAGCG	For intact CRISPR2-Cas systems	Variable	54.55	61.8
		**Reverse-** GCGCTACGTTCTGGGGATG			63.16	60.8
**9-**	**I-E-CR3**	**Forward-** GACGCTGGTGCGATTCTTGAG	For intact CRISPR3-Cas systems	Variable	57.14	66.0
		**Reverse-** CGCAGTATTCCTCAACCGCCT			57.14	66.0

### 3.3 Detection of the CRISPR/Cas system

The following primers were used for PCR to detect the frequency of CRISPR and associated Cas proteins:

### 3.4 Statistical analysis

The data were analysed using the following methods: mean, percentage, and frequency. The statistical software SPSS 20.0 was utilized for this objective to check if the correlations were significant statistically considering P value of 0.05 as statistical threshold. Chi-square analysis in addition to Fisher's exact test were used in the statistical analysis of this study.

## 4 Results

### 4.1 Susceptibility to antimicrobial agents

**Fig. 1:**
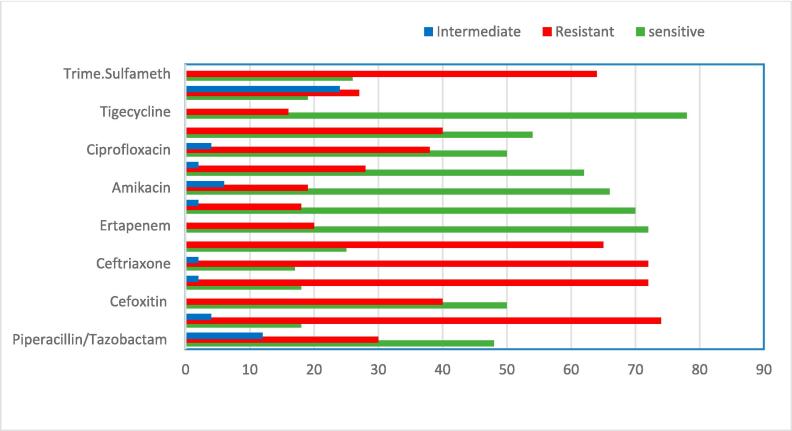
Antimicrobial susceptibility profiles of *K. pneumoniae* isolates are represented as percentages (MICs).

Various clinical specimens were used to isolate 100 isolates of *Klebsiella pneumoniae*. Fig. 1 reveals the antimicrobial susceptibility profiles conducted on all of the study isolates. Multiple antibiotics with distinct modes of action (those that impede cell wall production, DNA synthesis, permeability, and protein synthesis) were found to have a significant level of resistance in the isolates.

Several beta-lactam medications, such as carbapenems and beta-lactam/beta-lactamase inhibitors, were effective against most of the isolates. It is also common for patients to develop resistance to other medications, including aminoglycosides and fluoroquinolones. Only a small number of bacteria were sensitive to the four main antibiotic classes; the majority were resistant to cefazolin, ceftazidime, ceftriaxone, and cefepime, as shown in Fig. 1.

### 4.2 Characteristics of study isolates submitted the CRISPR loci findings

The Cas1, 3, 9, Cse1, CRISPR1, CRISPR2, and CRISPR3 genes were detected using PCR. Different patterns were observed in the 100 isolates depending on whether Cas and/or CRISPR genes were present. Our initial step was to check the chosen *K. pneumoniae* isolates for the presence of type I-E CRISPR1 genes. Since the Cas1 gene has been demonstrated to be crucial for the function of the CRISPR-Cas system, we employed primer sequences targeting this gene. According to Table 2, some isolates only carried CRISPR or Cas, in contrast to those who possessed both parts of the system. The presence of the system was found in 38 % of the study isolates. Various gene combinations were found to exist. The fact that 53 % of isolates had only CRISPR or Cas alone is remarkable. However, only nine isolates (9 %) were not harbour this system and associated cas protein as reflected in Table 2.

**Table 2:** PCR findings related to the detection of the CRISPR and associated cas encoding genes. The number of isolates featuring either gene or both is displayed, in addition to the number of isolates lacking genes.

**Note**: Cas 9, Cse 1, CRISPR 2/**Negative.**

**Fig. 2:**
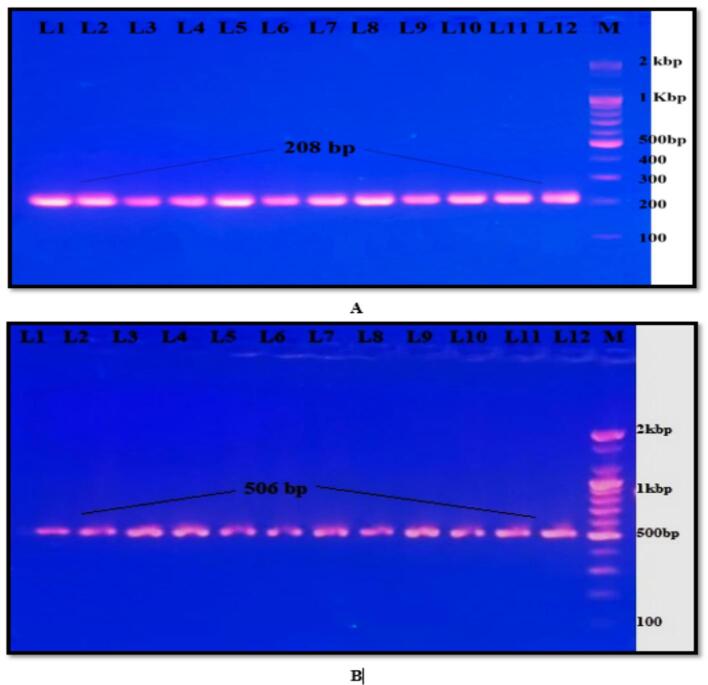
PCR products. A- Cas 1 gene with 208 bp. B- Cas 3 genes with 506 bp. M indicates the DNA marker (100 bp). The product was subjected to electrophoresis on 1.5 % agarose at a voltage of 5 V/cm.

Isolates of *Klebsiella pneumoniae* with representative gel images displaying cas1 and cas3 components were detected in 32 % and 18 %, respectively, of the samples, as shown in Fig. 2.

**Fig. 3:**
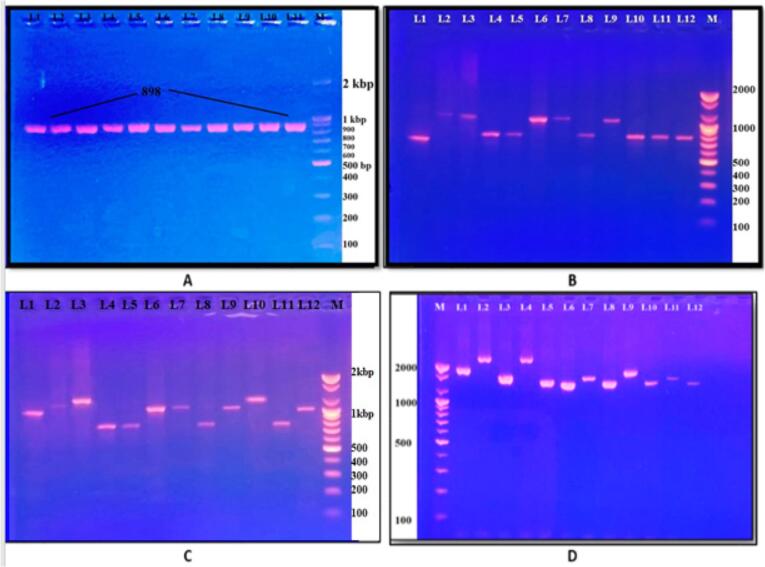
PCR products of A-CRISPR1 incomplete/genes (898 bp). B- PCR product of CRISPR1 intact genes (variable bp). C- CRISPR2 intact/genes (variable bp). D- PCR product of CRISPR3 genes (variable bp), typically generated via conventional PCR. An M represents a DNA marker that is 100 bases long. *The different K. pneumoniae* isolates harboring different CRISPR systems are represented by numerical characters. The product was subjected to electrophoresis on 1.5 % agarose at a voltage of 5 V/cm.

The primer I–E CRISPR1 localized on both sides of the CRISPR1 gene of *K. pneumoniae* (Essoh et al., 2013) was detected in approximately 78 % of the isolates, as shown in Fig. 3. A), for intact CRISPR-Cas systems, IE-CRISPR1 shows various bands depending on different spacers within the gene, which were detected in 27 % of the *K. pneumoniae* isolates (Fig. 3). For intact CRISPR-Cas systems, IE-CRISPR2 shows various bands depending on different spacers within the gene, which were detected in 34 % of the *K. pneumoniae* isolates (Fig. 3). C), and for intact CRISPR-Cas systems, IE-CRISPR3 shows various bands depending on different spacers within the gene, which were detected in 18 % of the *K. pneumoniae* isolates (Fig. 3.D).

### 4.3 Nosocomial *k. Pneumoniae* isolates involving the CRISPR and associated cas proteins and their correlations with resistance to selected antimicrobial agents

Seventy-one percent (71.0 %) of the isolates produced ESBL according to their antibiotic susceptibility and the findings of various phenotypic assays. The bacteria that produced ESBL were all resistant to more than one medication. In addition, metallo-β-lactamase (MBL) producers accounted for all 21 (21.0/%) of the isolates that exhibited carbapenem resistance (CR).

PCR analysis revealed that 32 (32.0 %) of the Cas1 PCR-positive isolates possessed a CRISPR array, whether it was CRISPR1, 2 and 3. The CRISPR/Cas system was prevalent in 27/71 (38.0 %) of the ESBL-producing isolates. Among the carbapenem-resistant bacteria, 6/21 (28.6 %) carried the CRISPR/Cas system, making them resistant to additional antibiotics (PDR or XDR), indicating an inverse relationship between the two variables. According to the data in Table 3.

**Table 3:** CRISPR/Cas Prevalence in ESBL-producing and carbapenemase-producing *K. pneumoniae* Isolates.

**Mechanisms of resistance**		**CRISPR/Cas**		**Total**
		**Positive No. (%)**	**Negative No. (%)**	
**ESBL**	**Positive**	27 (38.0 %)	44 (62.0 %)	71 (71.0 %)
	**Negative**	11 (38.0 %)	18 (62.0 %)	29 (29.0 %)
**Carbapenmase**	**Positive**	6 (28.6.0 %)	15 (71.4 %)	21 (21.0 %)
	**Negative**	32 (40.5 %)	47 (59.5 %)	79 (79.0 %)
**Total of ESBL**		38 (38.0 %)	62 (62.0 %)	100 (100.0 %)
**Total of Carbapenmase**		38 (38.0 %)	62 (62.0 %)	100(100.0 %)

The CRISPR/Cas system may help prevent drug resistance genes from being acquired since it is not very common in drug-resistant *Klebsiella pneumoniae*.

### 4.4 The correlation between the prevalence of the CRISPR and their associated cas proteins and resistance to antimicrobial agents

The antimicrobial resistance was more prevalent among *K. pneumoniae* isolates that were CRISPR/Cas-negative than among those that were CRISPR/Cas-positive. The following strains of CRISPR/Cas-resistant isolates were detected: piperacillin/tazobactam (10 %), cefazolin (30 %), cefoxitin (14.4 %), ceftazidime (30 %), ceftriaxone (30 %), cefepime (27.8 %), ertapenem (5.6 %), imipenem (4.4 %), amikacin (5.6 %), gentamicin (10 %), ciprofloxacin (14.4 %), levofloxacin (15.6 %), tigecycline (4.4 %), nitrofurantoin (8.9 %) and trimethoprim/sulfamethoxazole (26.7 %). Comparatively, 62.2 %, 23.3 %, 50 %, 30 %, 50 %, 50 %, 44.4 %, 15.6 %, 15.6 %, 15.6 %, 15.6 %, 15.6 %, 21.1 %, 26.7 %, 27.8 %, 11.1 %, 21.1 %, and 46.7 % of the CRISPR/Cas-negative isolates were resistant to these medications, respectively. Table 4 shows that among the isolates without the CRISPR/Cas system, resistant isolates are more likely to lack CRISPR, even if this difference is not statistically significant.

**Table 4:** Distribution of CRISPR/Cas positive and negative versus the resistance ratio among the selected antimicrobial agents used in this study considering P value is less than 0.05).

**Antimicrobial agents**	**Resistance (%)**		**P** **Value**
	**CRISPR/Cas (+) No. = 28**	**CRISPR/Cas (–) No. = 45**	
**Piperacillin/Tazobactam**	10.0 (9)	23.3 (21)	0.310
**Cefazolin**	30.0 (27)	50.0 (45)	0.429
**Cefoxitin**	14.4 (13)	30.0 (27)	0.389
**Ceftazidime**	30.0 (27)	50.0 (45)	0.429
**Ceftriaxone**	30.0 (27)	50.0 (45)	0.707
**Cefepime**	27.8 (25)	44.4 (40)	1.00
**Ertapenem**	5.6 (5)	15.6 (14)	0.246
**Imipenem**	4.4 (4)	15.6 (14)	0.151
**Amikacin**	5.6 (5)	15.6 (14)	0.310
**Gentamicin**	10.0 (9)	21.1 (19)	0.535
**Ciprofloxacin**	14.4 (13)	26.7 (24)	0.887
**Levofloxacin**	15.6 (14)	27.8 (25)	0.828
**Tigecycline**	4.4 (4)	11.1 (10.0)	0.555
**Nitrofurantoin**	8.9 (8)	21.1 (19)	0.309
**Trimethoprim/Sulfamethoxazole**	26.7 (24)	46.7 (42)	0.806

Based on our findings, resistant bacteria are more likely to have a CRISPR1 incomplete gene (on both sides of the CRISPR1 gene), followed by a CRISPR1 intact gene (n = 25; 25 %), CRISPR2 (n = 24; 24 %), and CRISPR3 (n = 13; 13 %). On the other hand, sensitive isolates without an MDR phenotype were more likely to have a CRISPR1 incomplete gene (n = 17; 17 %), CRISPR1 intact gene (n = 4; 4 %), CRISPR2 (n = 11; 11 %), or CRISPR3 gene (n = 5; 5 %).

The majority of the isolates in the Multidrug and extensively drug resistant groups appeared to be negative for the CRISPR/Cas system. The most striking result was that the CRISPR/Cas system appeared to be the most prevalent in MDR (no. = 23.0; 43.4 %) isolates, followed by the XDR (no. = 6; 31.5 %) and PDR (no. = 2.0; 22.2 %) isolates. However, when comparing CRs in their entirety (including XDR and PDR), a large number of these isolates (n = 20.0) were appeared to be deficient for the system of CRISPR and associated cas proteins as reflected in the Table 5.

**Table 5:** Correlations between the frequency of the CRISPR/Cas system and MDR, XDR, and PDR.

**MDR/XDR/PDR**	**Positive cases of CRISPR/Cas** **No. (%)**	**Negative cases of CRISPR/Cas** **No. (%)**	**Number of isolates**
**MDR^1^**	23 (43.4)	30 (56.6)	53 (53.0 %)
**XDR^2^**	6 (31.5)	13 (68.5)	19 (19.0 %)
**PDR^2^**	2 (22.2)	7 (77.8)	9 (9.0 %)
**CR ^3^**	8 (28.6)	20 (71.4)	28(28 %)

MDR: Multi-drug resistance; XDR: Extensively drug-resistance; PDR: Pandrug-resistance; ^1^All MDR isolates produced ESBLs; ^2^The XDR and PDR categories mainly comprised metallo-beta-lactamase producers (CRs) while ^3^represent the combination of PDR and XDR.

**Fig. 4:**
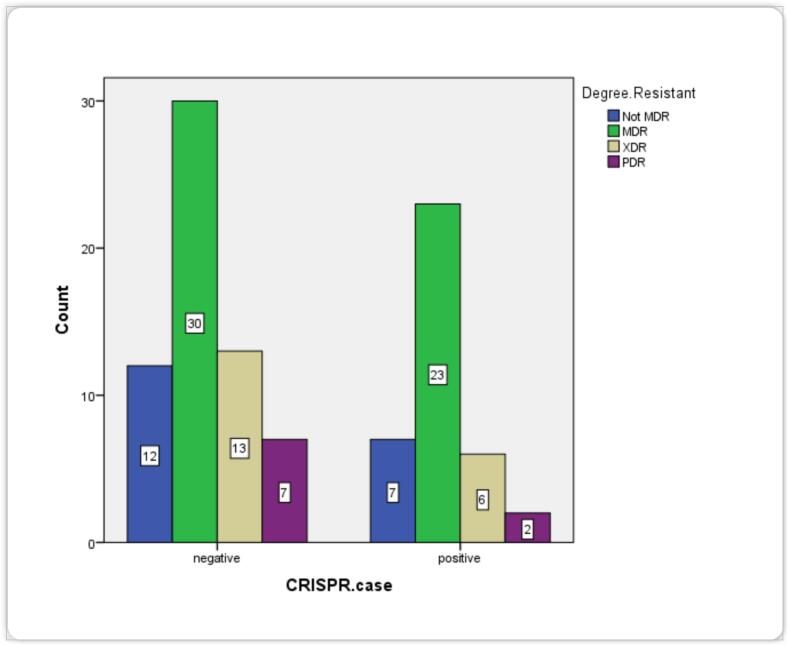
The inverse correlation between the absence or presence of CRISPR/Cas system and the levels of antimicrobial resistance among not-MDR, MDR, XDR, and PDR *Klebsiella pneumonia.*

Fig. 4 reveals that the MDR and XDR groups had the most substantial variances, with the majority of these strains being negative for CRISPR/Cas systems. The difference between non-MDR and PDR was not clear due to the small total number of strains per group, which reduced the ability of appropriate statistical comparisons. The inverse correlation between the absence or presence of the CRISPR/Cas system and the degrees of antibiotic resistance among non-MDR, MDR, XDR and PDR *Klebsiella pneumoniae*.

## 5 Discussion

Recently, the healthcare institutions including hospitals have become recognized as the main reservoirs for a range of pathogenic bacteria that are resistant to drugs (MDR), including *K. pneumoniae* species (Alduhaidhawi et al., 2022). The factors causing this phenomenon is the unrestrained and uncontrolled of the antimicrobial agents used for the treatment of infections, which forces bacteria to develop resistance. The spread of microorganisms that are resistant to antibiotics from healthcare facilities, such as hospitals, to the general population is a true problem (Samreen, 2021).

The majority of penicillins and third-generation cephalosporin medicines do not work against bacteria that produce ESBLs. The primary bacteria that cause ESBLs are *Klebsiella* species and *Escherichia coli*. According to an update in the Centers for Disease Control and Prevention, ESBL-producing Enterobacteriaceae cause approximately 26,000 healthcare-associated illnesses annually (Kim et al., 2015). In comparison to bacteraemia patients caused by Enterobacteriaceae isolates which are not harbour ESBLs, those which caused by ESBL-producer isolates had an approximately of 57.0 % increased risk of death (Chopra et al., 2015).

To identify the connection between the system of CRISPR and associated cas proteins and antimicrobial resistance, our study revealed that the antibiotic resistance included the study clinical isolates of *K. pneumoniae* with various resistance status. According to a previous study, *K. pneumoniae* has a notably high frequency of antibiotic resistance to several significant antimicrobial drugs (Al-Kubaisy et al., 2020) The findings of the study indicated a significant rate of resistance to β-lactam antibiotics, including ceftriaxone (88.2 %), ceftazidime (82.3 %), and cefepime (52.9 %). This might occur because people use these medicines frequently without proper medical care. According to Ejaz and colleagues (Ejaz et al., 2013), the resistance to third-generation cephalosporin was detected in 88.63 % of the study isolates of *K. pneumoniae*.

The findings of numerous national and international studies are consistent with the high percentage of resistant isolates. These findings paralleled those of Khalaf and Al-Ouqaili (Khalaf and Al-Ouqaili, 2018), who reported resistance rates to ceftazidime and ceftriaxone 84 % and 88 % respectively. In a study of Al-Kubaisy and colleagues, they documented that resistant to ceftriaxone (88.2 %) and ceftazidime (82.3 %), respectively, were *K. pneumoniae* bacteria (Al-Ouqaili et al., 2020b). Compared to what Gharavi and associates who reported that the investigation revealed a greater percentage of 3rd generation cephalosporins resistant isolates of *K. pneumoniae*; among the isolates tested in northwest Pakistan, 54.35 % revealed resistance to ceftriaxone (Gharavi et al., 2021). Al-Ouqaili and colleagues (Khalaf and Al-Ouqaili, 2018) concluded in his study that 92 % of study isolates of *K. pneumoniae* appeared resistant to ceftazidime while 96 % of them were cefotaxime resistant.

Because of the extensive usage of these antimicrobial agents in recent period, enterobacterial isolates have acquired resistance to fluoroquinolones. According to Ahmadi et al. (Jomehzadeh et al., 2022), *K. pneumoniae* is the Enterobacteriaceae member that is most prone to developing resistance to quinolones, among other antibiotics. This multidrug-resistant opportunistic bacterium poses a serious challenge to medical professionals treating infectious diseases worldwide. Among the antibiotics tested, the most common were ceftriaxone (73.0 %) and cefepime (66.0 %), whereas the percentages of MDR and XDR isolates were 53 % (n = 53) and 19 % (n = 19, respectively).

The study revealed that 40.0 % and 43.0 % of the study isolates of *K. pneumoniae* were resistant to ciprofloxacin and levofloxacin respectively. These results disagreed with those of Jomehzadeh et al. (Jomehzadeh et al., 2022), who documented varying RRs for ciprofloxacin (18.5 %) and levofloxacin (30.4 %).

Antibiotics have transformed the treatment of numerous infectious diseases; however, the public's access to these drugs over the counter, along with their increasing usage, which includes errors in dosage and treatment duration as well as careless and inaccurate prescribing, has led to an increase in antibiotic resistance (Alhamdany, 2018). It was first suggested that specific bacterial genotypes could be eradicated by using CRISPR/Cas system as an antimicrobial agent (Bikard et al., 2012).

Recently, the number of investigations revealed that the CRISPR/Cas system can precisely remove genes linked to drug resistance from populations of bacteria and can re-sensitize bacteria to antibiotics by eradicating plasmids that encode antimicrobial resistance (AMR) genes. Several issues need to be resolved before CRISPR-Cas can be used to target antimicrobial resistance in microbial communities in the nature. Finding a workable distribution plan will be crucial if this technology is to realize its full potential for limiting the ecological and therapeutic dissemination of AMR caused by mobile genomic elements (MGEs). The efficiency of this process can be greatly enhanced by reprogramming simple CRISPR/Cas constructs to target specific genes of interest. This finding might preserve or reverse the antibacterial activity of antibiotics in addition to helping to combat reservoirs of antimicrobial resistance. The CRISPR/Cas antimicrobial selectivity was demonstrated by isolating individual bacterial isolates from a population of mixed *Escherichia coli* genotypes through the introduction of a plasmid expressing CRISPR/Cas that targeted a particular sequence unique to each genotype. Two investigations have demonstrating the feasibility of delivering CRISPR-Cas9 via phagemids, which are plasmids encased in phage capsids, to eradicate two clinically important bacterial infections: *Staphylococcus aureus* and *Escherichia coli*. One of these studies successfully eradicated plasmids carrying AMR genes from bacteria by delivering CRISPR/Cas9 constructs tailored to target these genes via phagemid transduction. In addition, bacteria with chromosomal AMR genes were engineered to express CRISPR/Cas9 using conjugative plasmids (Citorik et al., 2014). Another experiment demonstrated the successful removal of plasmids carrying AMR genes and the successful resensitization of bacteria to antibiotics through the use of sequence-specific CRISPR/Cas9 gene delivery to pathogenic bacteria (Pursey et al., 2018).

Zhou and colleagues (Zhou et al., 2022) focus on the Klebsiella CRISPR-Cas system to investigate the interaction between mobile genetic components and CRISPR as well as the different factors influencing CRISPR. According to the investigation, CRISPR both hinders and defends against foreign mobile devices. It is also possible that some genes and mobile genetic elements had a major role in the development and evolution of CRISPR.

We detected a variety of CRISPR/Cas system characteristics in our isolates during our investigation. Because CRISPR arrays are exposed to varying bacteriophages throughout their lifecycle, their quantity, sequence, and size are highly variable. This finding was compatible with the findings of Koonin and Makarova (Koonin and Makarova, 2019), who reported that numerous species of bacteria display very different patterns, quantities, and durations of their CRISPR arrays. Kuno and colleagues demonstrated that the Microcystis aeruginosa genome has a diverse array of antiviral defense systems, which is compatible with its high cyanophage diversity (Kuno et al., 2012).

CRISPR/Cas systems have been linked to the production of virulence factors in bacteria as well as the restriction of foreign DNA entry into those organisms. Few studies have shown the in vivo action of these systems, although they have been extensively examined in a variety of taxa, including pathogens and nonpathogens. Therefore, it is difficult to locate information in publications about spacer content, such as the percentage of spacers that match a known sequence or are exclusive to a certain species, according to Bondy-Denomy and Davidson (Bondy-Denomy & Davidson, 2014).

Articles that would offer a basic perspective on the operation of this system would include details regarding the spacers' contents, the percentage of those that match a previously identified sequence, or those exclusive to a particular species. The interpretation of these systems' roles in the isolates under study will benefit from these data, which should further our knowledge of bacterial evolution and the effects of horizontal gene transfer on human health and the environment. To the best of our knowledge, *K. pneumoniae*, one of the top five pathogens causing nosocomial infections globally, has not yet been thoroughly investigated or characterized. It is a member of the ESKAPE group (Djordjevic et al., 2012). Due to its large plasmids, virulence factors, and ease of dispersal in hospital wards, *K. pneumoniae* has various ecological advantages that allow it to adapt to a variety of habitats (El Fertas-Aissani et al., 2013). Consequently, we are interested in determining whether *K. pneumoniae* has CRISPR/Cas systems and, if so, how these systems are associated with virulence, multidrug resistance, horizontal gene transfer, and other attributes. Researchers have found that the CRISPR/Cas system, which uses spacers derived from invading elements to control the immune response in a sequence-specific way, protects prokaryotes from viruses (Barrangou and Horvath, 2012). As a result, the spacer repertoire might represent the lifestyle of bacteria (Ilıkkan and Özge, 2022).

Up to our knowledge, this is the first study from the Arab world that focused on the CRISPR-Cas system and its link to MDR, XDR, and PDR *K. pneumoniae* strains, which are exceedingly difficult, if not impossible, to treat with current antibiotics. A total of 38.0 % (38/100) of the *K. pneumoniae* isolates in this investigation exhibited CRISPR/Cas, which is regarded as a low proportion. In contrast, CRISPR1/Cas, CRISPR2/Cas, and CRISPR3/Cas were present in 38.0 %, 34.0 %, and 18.0 % of the isolates, respectively. Most of the isolates that were tested were determined to be negative for CRISPR/Cas systems because they were resistant to numerous antibiotics, which may have contributed to the low incidence of these systems in this study. When contrasting our results to those of other researchers, Li and colleagues (Li et al., 2018) who reported 30.7 % of clinical *K. pneumoniae* isolates in Taiwan possessed the CRISPR/Cas system when we compared our results to those of previous investigations. Wang et al.'s study (Wang et al., 2020a) revealed that 21.32 % (29 out of 136) of *K. pneumoniae* isolates possessed CRISPR/Cas. The remaining isolates tested positive for either CRISPR2 (n = 13) or CRISPR3 (n = 2), with 14 isolates testing positive for both genes. Zhou and colleagues (Zhou et al., 2022) reported that approximately one-third of 300 *K. pneumoniae* isolates exhibited this mechanism. Comparatively, according to a recent study by Lin et al., only 6 out of 52 *K. pneumoniae* isolates have this system (Lin et al., 2016). For this reason, it appears that *K. pneumoniae* does not have a widely distributed CRISPR/Cas system.

The strengths of the present study are based on its results, which showcase the current discoveries of the CRISPR/Cas system and their correlation with the lack of antibiotic resistance genes. It seems that *K. pneumoniae* does not have a broadly distributed CRISPR/Cas system, as the system was fully viable for isolates with either whole or tentative genomes (Ostria-Hernández et al., 2015). Clinical isolates of *K. pneumoniae* isolated from healthcare facilities showed evidence of CRISPR/Cas in 33 out of 100 (33.0 %). The CRISPR/Cas system incidence was from 30.7 % (54/176) to 12.4 % (27/217) (Lin et al., 2016).

There is an inverse relationship between antibiotic resistance to the various antimicrobial drugs utilized and the presence of the CRISPR/Cas system. According to our findings, all of the isolates that were subjected to this system exhibited decreased resistance to aminoglycosides, quinolones, and β-lactams/enzyme inhibitors. A research laid down by Mackow et al. (2019) concluded that most *K. pneumoniae* isolates carrying CRISPR/Cas were pan sensitive, while isolates lacking CRISPR showed signs of resistance to several drugs. According to Wang and colleagues (Wang et al., 2020a), the existence of this system may be linked to reduced drug resistance and, in certain cases, may stop *K. pneumoniae* from acquiring drug resistance genes. According to Lin et al. (Lin et al., 2016), the presence of CRISPR/Cas loci was significantly associated with carbapenem resistance in *K. pneumoniae*. Evidence of a negative correlation between CRISPR/Cas and antibiotic resistance was suggested by Gholizadeh and colleagues (Gholizadeh et al., 2020b), who reported that *K. pneumoniae* isolates that were developed via the CRISPR/Cas system were less resistant to aminoglycosides, quinolones and inhibitors of β-lactams.

Resistant bacteria harboring CRISPR or Cas genes may indicate that these systems were once prevalent but transitioned out of function. They are eliminated to allow the bacteria to acquire AMR genes and develop resistance (Pinilla-Redondo et al., 2022). Because this study was cross-sectional, there were few susceptible bacteria, and the majority of the bacteria responsible for these diseases were MDR, XDR, or PDR, which is highly worrisome.

Notably, the lowest ratio of the presence of the CRISPR/Cas system was observed in PDR (resistant to all antimicrobial agents used), while a higher CRISPR/Cas system ratio was observed in MDR isolates. This result supported the hypotheses that there is an inverse correlation between the presence of the CRISPR/cas system and antibiotic resistance (Jwair and colleagues (Jwair et al., 2023. Furthermore, most strikingly, as the degree of antibiotic resistance increases (from MDR to XDR to PDR), the frequency of the CRISPR-Cas system decreases. This may be because the CRISPR system protects the *K. pneumoniae* genome against invasion by genetic elements carrying antibiotic resistance genes to maintain genetic homeostasis. Furthermore, foreign genetic plasmids and other conjugative ones, may harbour beneficial genes which may increase bacterial fitness, like virulence and antimicrobial resistance, in the environment.

It was concluded that CRISPR/Cas systems can protect against exogenous antibiotic resistance. There is a low frequency of these systems in study isolates that are resistant to these antibiotics, at which point this type of resistance might develop when CRISPR/Cas system elements are missing. This is crucial because these systems could eventually be developed as weapons against bacteria which are antibiotic resistant. New hope for the eradication of MDR isolates has emerged with the development of the CRISPR/Cas system, which employs RNA-guided DNA nuclease to target bacterial genes and render antibiotic-resistant cells more susceptible to drugs.

## CRediT authorship contribution statement

**Hekmat A. Owaid:** Methodology, Investigation, Funding acquisition, Formal analysis. **Mushtak T.S. Al-Ouqaili:** Writing – review & editing, Writing – original draft, Supervision, Project administration, Data curation, Conceptualization.
